# Single-molecule correlated chemical probing reveals large-scale structural communication in the ribosome and the mechanism of the antibiotic spectinomycin in living cells

**DOI:** 10.1371/journal.pbio.3000393

**Published:** 2019-09-05

**Authors:** Arnab Sengupta, Greggory M. Rice, Kevin M. Weeks

**Affiliations:** Department of Chemistry, University of North Carolina Chapel Hill, Chapel Hill, North Carolina, United States of America; Yale University, UNITED STATES

## Abstract

The ribosome moves between distinct structural states and is organized into multiple functional domains. Here, we examined hundreds of occurrences of pairwise through-space communication between nucleotides in the ribosome small subunit RNA using RNA interaction groups analyzed by mutational profiling (RING-MaP) single-molecule correlated chemical probing in bacterial cells. RING-MaP revealed four structural communities in the small subunit RNA, each distinct from the organization defined by the RNA secondary structure. The head domain contains 2 structural communities: the outer-head contains the pivot for head swiveling, and an inner-head community is structurally integrated with helix 44 and spans the entire ribosome intersubunit interface. In-cell binding by the antibiotic spectinomycin (Spc) barely perturbs its local binding pocket as revealed by the per-nucleotide chemical probing signal. In contrast, Spc binding overstabilizes long-range RNA–RNA contacts that extend 95 Å across the ribosome that connect the pivot for head swiveling with the axis of intersubunit rotation. The two major motions of the small subunit—head swiveling and intersubunit rotation—are thus coordinated via long-range RNA structural communication, which is specifically modulated by Spc. Single-molecule correlated chemical probing reveals trans-domain structural communication and rationalizes the profound functional effects of binding by a low–molecular-mass antibiotic to the megadalton ribosome.

## Abstract 2

Single molecule chemical probing of pair-wise interactions across the ribosome in living cells redefines the domains of the small subunit of the ribosome and reveals that the antibiotic spectinomycin disrupts ribosome function by over-stabilizing interactions that span nearly 100 Å.

## Introduction

The ribosome is a megadalton complex that undergoes two large-scale motions during translation [1,2]. First, the two ribosome subunits rotate relative to each other in a ratchet-like manner. The bacterial ribosomal small subunit rotates by 6°–9° relative to the large subunit during translocation, pivoting about an intersubunit bridge, termed B3 [3–6]. B3 is unique among the intersubunit bridges (termed B1–B8, with several subtypes) in that it remains intact during intersubunit rotation whereas other bridges undergo conformational changes during dynamic cycles of disruption and reformation [5,7]. A second large-scale movement, small-subunit head-domain swiveling, accompanies translocation and involves pivoting at two “hinge” sites [6,8–10]. The antibiotic spectinomycin (Spc; molecular weight = 332 g/mol) is thought to freeze the head domain in a partially swiveled state that blocks translation [8,11,12], emphasizing the importance of this movement during translocation for ribosome function.

Past investigations have inferred features of ribosome dynamics during translocation by visualizing stable and semistable intermediate states using high-resolution approaches [5,6,13–15]. In addition, the dynamics of specific pair-wise elements have been examined by single-molecule approaches [15,16]. These studies revealed that the ratchet-like intersubunit rotation of the ribosomal subunits occurs contemporaneously with the swiveling motion of the head domain of the small ribosomal subunit [8,9]. However, structural communication between head swiveling and intersubunit rotation within the ribosome is not well understood, and internal ribosome motions have not been analyzed in living cells. Moreover, although ribosome activity can be strongly affected by low–molecular-mass antibiotics, mechanistic understanding of how small-molecule binding influences global ribosomal RNA (rRNA) dynamics is limited.

**Fig 1:**
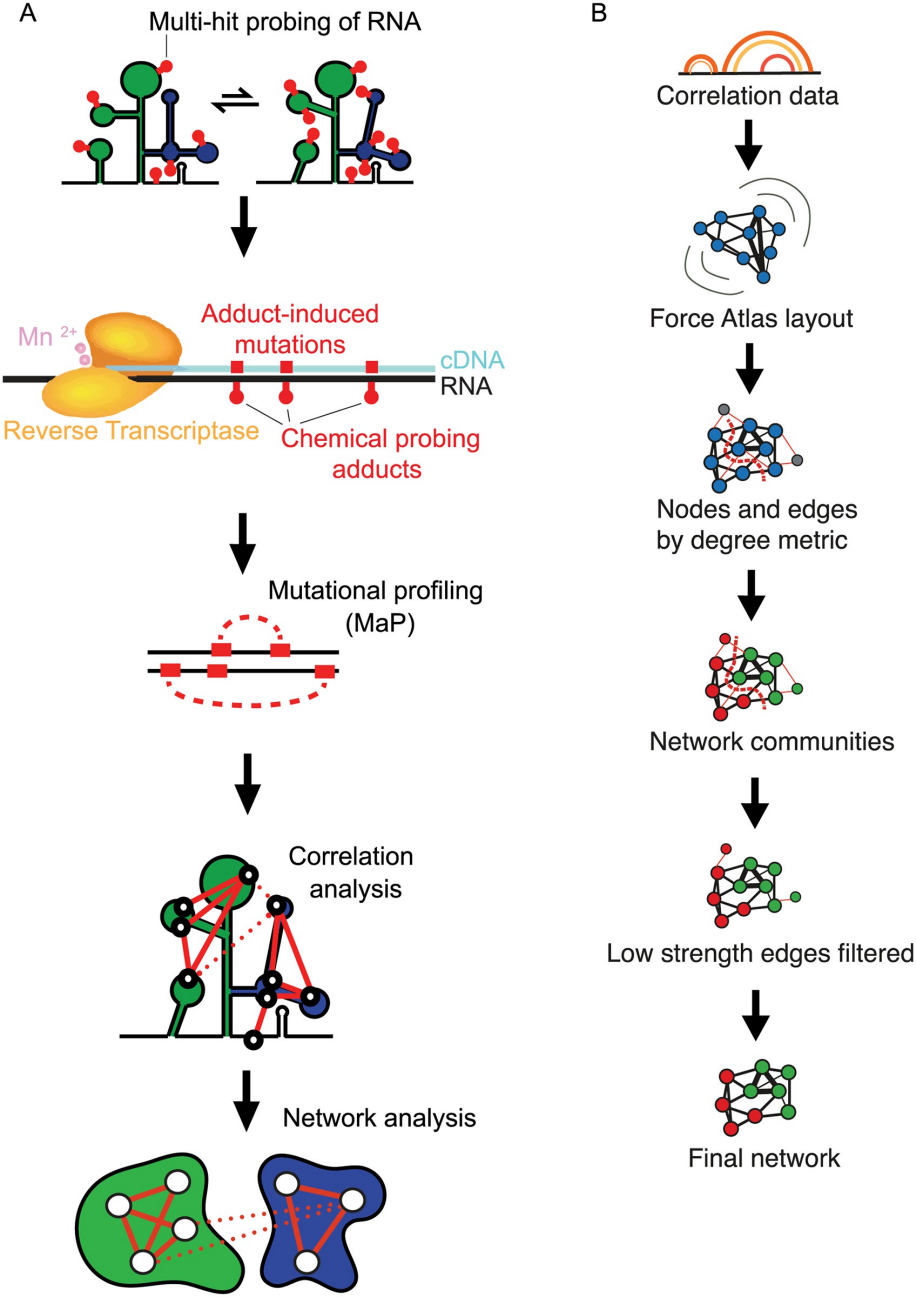
RING-MaP single-molecule correlated chemical probing and network analysis of a large RNA. (A) Overview of the RING-MaP experiment and network analysis. DMS chemical adducts on an RNA are detected by MaP, which records adduct sites as mutations and indels in the cDNA sequence generated by reverse transcription. Mutations co-occurring on the same read are analyzed for correlations that report RINGs, which are then visualized using a network graph. (B) Workflow for network analysis and detection of structural communities. Network analysis was performed using *Gephi*. DMS, dimethyl sulfate; MaP, mutational profiling; RING, RNA interaction group; RING-MaP, RNA interaction groups analyzed by mutational profiling.

We recently developed the RNA interaction groups analyzed by mutational profiling (RING-MaP) chemical probing strategy that makes it possible to detect multiple chemical modification events on the same strand of RNA using massively parallel sequencing and to analyze these events for correlations (Fig 1A) [17]. The RING-MaP experiment is thus a single-molecule recording of co-occurring modifications in the same RNA molecule. By analyzing correlations between chemical modification events, it is possible to measure the through-space structural communication between nucleotides and to group these interactions in network communities (Fig 1B) [17–20]. A variety of chemical probes, including dimethyl sulfate (DMS), penetrate cell membranes and can be used to interrogate RNA structure in living cells. Here, we used RING-MaP with DMS to characterize hundreds of occurrences of through-space internucleotide communication in the 16S rRNA in *Escherichia coli* cells to define structural communities within the ribosome small subunit and to examine the effects of binding of the antibiotic Spc on ribosome structural dynamics.

## Results

### Multisite chemical probing of the 16S rRNA in living *E*. *coli* cells

*E*. *coli* cells in the mid-log phase of growth were treated with DMS under three conditions: unperturbed, treated with rifampicin (Rif), or first treated with Rif and then with Spc. Rif treatment inhibits transcription by RNA polymerase and allows the 30S ribosomal subunit to assemble into stable, fully formed complexes [21–23]. Rif treatment reduces intermediate states of ribosome assembly to a small fraction of the total population of ribosome complexes; subsequent Spc treatment thus has minimal impact on ribosome assembly [22]. Rif also promotes degradation of polysomes [24], and pre-treatment with Rif reduces subsequent Spc-induced accumulation of polysomes [25]. After DMS treatment, the 16S rRNA was purified and subjected to mutational profiling (MaP). MaP is a high-throughput sequencing technology that enables detection of sites of chemical modifications in an RNA strand as internal sequence changes in a cDNA synthesized during reverse transcription [17,26] (Fig 1A). In this work, we developed new experimental conditions to allow efficient generation of long sequencing reads despite a high level of chemical modification and to detect through-space correlated nucleotide reactivities over distances spanning roughly 500 nucleotides. We also developed a new algorithm for detecting correlated nucleotide reactivities for randomly primed data over long sequence distances (Methods and S1 Fig). DMS reactivity is not strongly correlated with solvent accessibility (*R*^2^ = 0.007), and DMS reacts broadly with the 16S rRNA, affording good coverage of structural communities across the RNA.

### Profound differences in chemical probing data as assessed by per-nucleotide versus correlated reactivities

**Fig 2:**
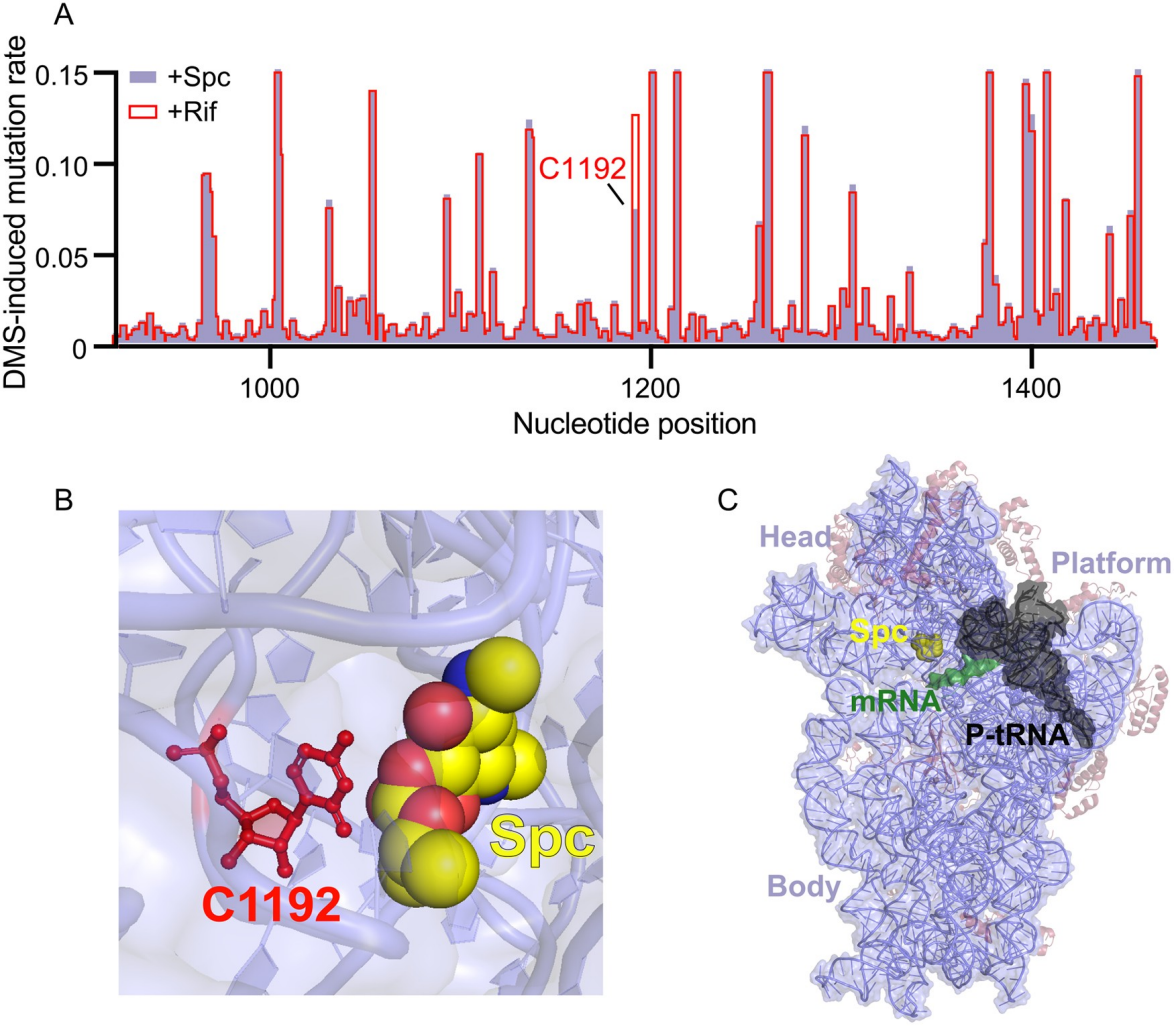
Spc binding site in the 16S rRNA in *E*. *coli* cells revealed by DMS footprinting. (A) Per-nucleotide DMS-induced mutation rate profiles for 16S rRNA in cells treated with Rif (+Rif) and with Rif and Spc (+Spc). The plot shows a 550-nucleotide region spanning the 3' domain of the 16S rRNA; a single nucleotide at C1192 had a notable difference between the two experiments. The underlying data for this figure are available at https://doi.org/10.6084/m9.figshare.9252995.v1. (B) Model of Spc bound near C1192 (in red) of the 16S rRNA. (C) Structure of the small ribosomal subunit. 16S rRNA is light purple, ribosomal proteins are brown, mRNA is green, tRNA is black, and Spc is yellow. 30S subunits domains are labeled by morphology. Ribosome structure in all figures are from PDB 4v56 and 5afi [5,12]. DMS, dimethyl sulfate; PDB, Protein Data Bank; Rif, rifampicin; rRNA, ribosomal RNA; Spc, spectinomycin.

**Fig 3:**
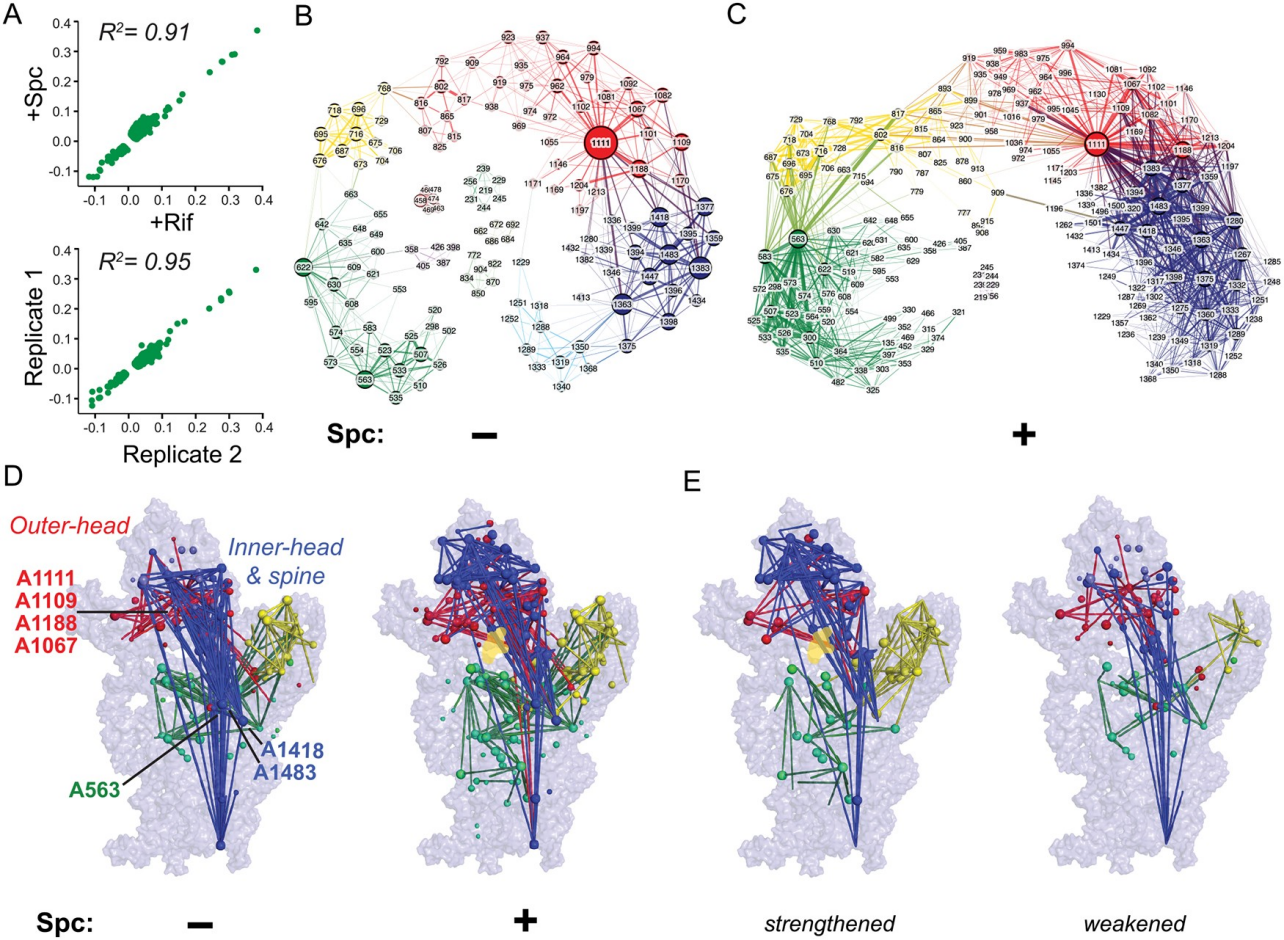
Network analysis applied to RING-MaP correlation data reveals that Spc induces extensive changes in through-space structural communication in the ribosome. (A) Correlation of RING-MaP data for independent experiments based on Spc-untreated versus Spc-treated samples (top panel; both samples pretreated with Rif); and between biological replicates (bottom panel) (B–C) Network analysis of structural communication in the 16S rRNA in (B) the absence and (C) the presence of Spc. Network analysis reveals four structural communities (blue, red, yellow, and green). Nodes are colored by community and are sized based on the number of correlations with other nodes in the network. Edges indicate internucleotide correlations, and edge weights indicate correlation strength. For both–Spc and +Spc conditions, cells were pretreated with Rif, which allows the rRNAs to fully assemble into complete subunits [22]. (D) Superposition of network nodes on the three-dimensional structure of the 30S subunit. Nodes were categorized by strength into strong, medium, and weak indicated by large, medium, and small spheres, respectively; strong interactions are shown as colored lines. (E) Edges that were strengthened (left) or weakened (right) upon addition of Spc. The underlying data for this figure are available at https://doi.org/10.6084/m9.figshare.9252995.v1. Rif, rifampicin; RING-MaP, RNA interaction groups analyzed by mutational profiling; rRNA, ribosomal RNA; Spc, spectinomycin.

In the simplest interpretation, MaP data can be used to generate straightforward reactivity versus position profiles, analogous to data obtained in conventional chemical probing experiments. We examined the effect of Spc binding to the small subunit by comparing the in-cell chemical probing signal in samples treated with Rif (+Rif) with that of samples treated with Rif and Spc (+Spc). Under the conditions employed in this study, DMS reacts primarily with A and C nucleotides that are (at least transiently) accessible at their base pairing face. As assessed by in-cell probing, Spc binding protected a single site in the 16S rRNA, C1192 (Fig 2A). Protection at C1192 is consistent with prior footprinting experiments with DMS [27–29] and with high-resolution structures of the *E*. *coli* ribosome complexed with Spc, which show that Spc binds in the minor groove of helix h34 (Fig 2B and 2C) [11,12]. We did not observe DMS-mediated protection at C1063, as observed previously using purified ribosomes [28], because DMS does not react with this nucleotide in the cellular environment in the absence of Spc. In addition, the overall near-identical per-nucleotide DMS reactivities of the +Rif and +Spc samples revealed that the structure of the in-cell 30S subunit and average association with the 50S subunit and with translation factors was similar in the presence and absence of Spc (Fig 3A). Overall, the per-nucleotide DMS chemical probing data suggest that Spc binding induces little change to ribosome structure in cells and that alterations are limited to its localized binding site.

The MaP strategy also allows chemical probing data to be analyzed to detect correlated chemical events between any two nucleotides on a single strand (Fig 1A). We refer to correlated chemical modification reactions as RNA interaction groups (RINGs). RINGs report through-space structural communication in RNA [17]. We obtained two full biological replicates for 16S rRNA probed under two states: in unperturbed cells and for *E*. *coli* grown in +Rif and the +Spc conditions using the RING-MaP strategy; correlations between biological replicates were high (Fig 3A). RING correlation networks were identified in each biological replicate and then merged into a single dataset containing correlations that occurred in both replicates (S2 Fig). RING correlations were not appreciably different between the untreated and +Rif cells (S2 Fig), suggesting that under both conditions most ribosomes are fully assembled. In the +Rif condition, many RING correlations were relatively weak (Fig 3B), consistent with correlations that originate from an averaged ensemble of cellular ribosomes in different conformations.

We next examined correlations for in-cell ribosomes probed after addition of Spc to the fully assembled ribosomes. In-cell treatment with Spc caused extensive and profound changes in through-space correlations across the length of the 16S rRNA, with especially strong enrichment in correlation density at the 5' and 3' ends of the RNA (Fig 3B and 3C, S3 Fig). Based on prior work, the averaged ensemble of cellular ribosomes in the +Spc condition would include Spc-arrested ribosomes that were trapped in an intermediate state of tRNA translocation, also bound by elongation factor-G [15]. Many correlations are shared between the +Rif and the +Spc states. However, in the presence of Spc, many correlations already present in the +Rif state were strengthened, and new correlations were observed (Fig 3C, S3 Fig). The observed changes in the number and strength of RING correlations are consistent with Spc-mediated inhibition of swiveling of the head domain.

To summarize, in strong contrast to a conventional interpretation of chemical reactivity on a per-nucleotide basis, as a function of position, which suggested little change in ribosome structure (Fig 2), the correlated chemical probing experiment revealed that Spc induces extensive and profound global changes in the 30S ribosome subunit (Fig 3, S3 Fig). The RING experiment detected extensive through-space interactions throughout the 16S rRNA and revealed dramatic changes in through-space interactions upon addition of Spc. The extensive through-space structural communication revealed in our study reflects both changes in higher-order intramolecular RNA–RNA tertiary structure interactions and also structural communication modulated by contacts with neighboring rRNA, translation factors, and other proteins.

### 30S domain architecture as revealed by network communities

The RING-MaP experiment identified many cases in which one nucleotide interacts with several other nucleotides in the 16S rRNA as revealed by mutual correlations in chemical reactivity patterns. We analyzed the correlated chemical reactivity RING data as a network graph with nucleotides represented as nodes and correlation strengths between nucleotides as edges (Fig 1B) [30]. The network graph representation of RING data allowed us to identify strong nodes and correlations and to group nucleotides into communities in an unbiased way. Network analysis divided the internucleotide communities in the 16S rRNA into 4 groups (Fig 3). This representation also allowed us to evaluate global changes in through-space RNA structural communication upon ligand binding.

**Fig 4:**
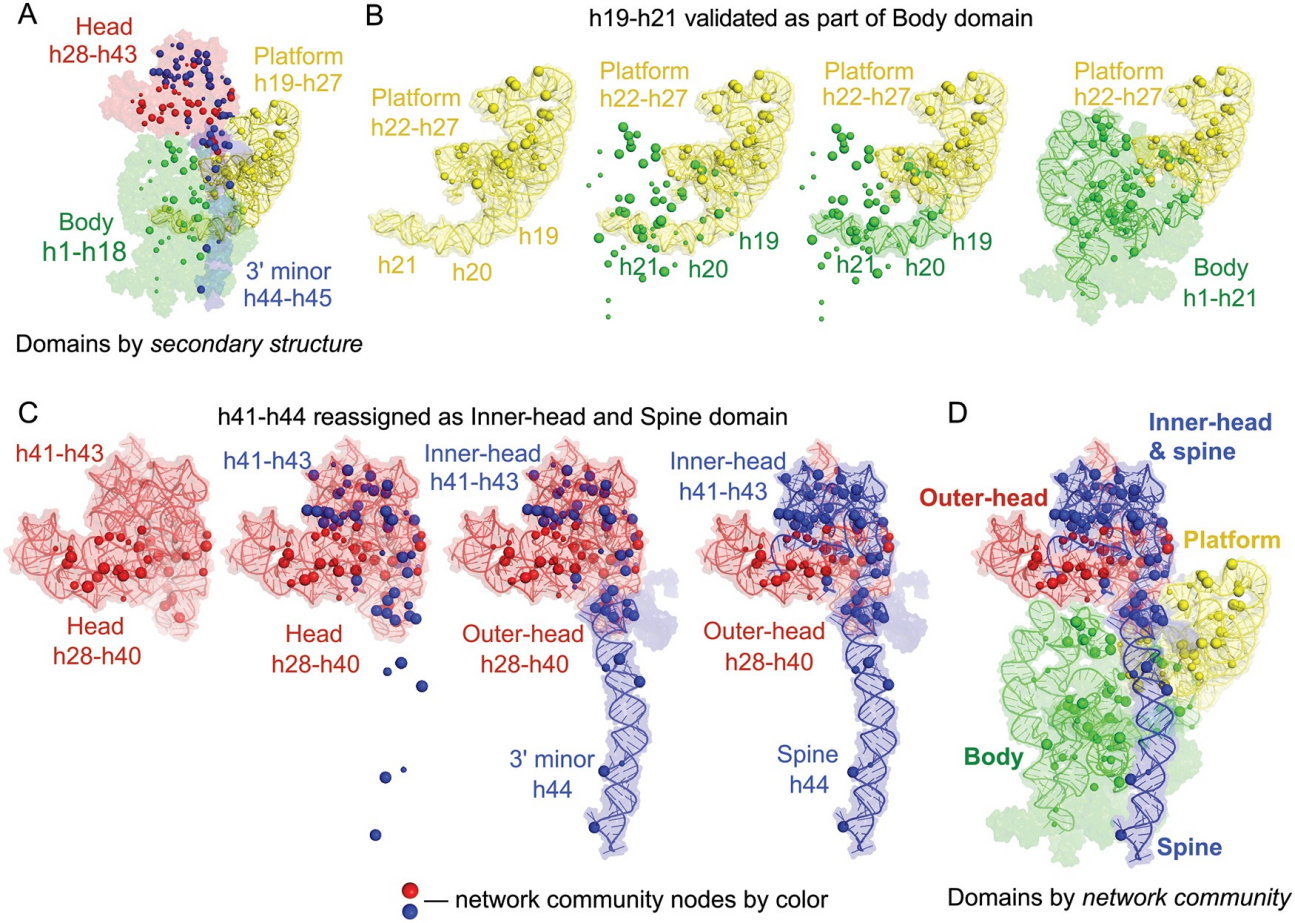
Domain architecture of the 16S rRNA as defined by network communities. Spheres indicate strong network nodes, colored by community. (A) 16S rRNA domain architecture based on secondary structure. Note that part of the central (platform) domain, as defined by the 16S rRNA secondary structure, is a structural component of the body. (B) Network communities correctly define the body domain and the more compact platform domain (yellow). These domains emerged naturally from the network analysis, even though no three-dimensional structural information was included in the network analysis. (C) The conventional head domain contains two RING network communities occupying distinct regions, defined here as the outer-head domain (red) and the inner-head and spine domain (blue). (D) Small-subunit domain model based on RING network communities in the 16S rRNA. The domains shown correspond to RING-MaP network community nodes of high correlation observed for the +Spc (+Rif) treatment condition; however, the same communities are observed in the absence of Spc. Rif, rifampicin; RING, RNA interaction group; RING-MaP, RNA interaction groups analyzed by mutational profiling; rRNA, ribosomal RNA; Spc, spectinomycin.

The 16S rRNA contains four domains based on the organization of the secondary structure of the RNA, conventionally termed the 5', central, 3'-major, and 3'-minor domains. These secondary structure domains also largely overlap with the physical structure of the 30S subunit such that the 5', central, and 3' RNA secondary structure domains correspond approximately to the body, platform, and head structures, respectively, and the 3'-minor domain forms an extended helix (h44) that extends from the head across the body (Fig 4A) [12].

When we superimposed strong nodes identified in our network analysis of the 16S rRNA on the 30S subunit structure, we observed that the four RNA communities are roughly centered on the body (green), platform (yellow), and head (red and blue) domains in 30S subunit (Fig 4A). This is a satisfying and important result because we did not impose the conventional domain organization on the network analysis. We can detect RING interactions over approximately 500 nucleotides, which would have allowed us to detect an alternative long-range organization of the rRNA.

The RING data correctly detected an important nuance of the domain organization of the small subunit. Helices 19, 20, and 21 are part of the central domain as defined by RNA secondary structure (Fig 4B). The central domain largely overlaps with the platform of the 30S subunit; however, helices 19–21 contain multiple nodes assigned to the green community (Fig 4B). RING analysis thus correctly assigned helices 19, 20, and 21 to the body domain (Fig 4B, green). The platform domain in the RING-directed model contains only helices 22 to 27 (Fig 4B and 4D, yellow). This difference in domain organization between the established 16S rRNA secondary structure and domain organization in three-dimensional space was identified in the first crystal structure of the 30S subunit [31]. Strikingly, this same nonintuitive organization emerged naturally from our network analysis. Thus, network analysis based on single-molecule correlated chemical probing can correctly identify structurally distinct and cohesive structural domains.

### RING analysis reveals a new domain organization for the 30S subunit

RING-based networks also revealed two major discrepancies with the conventional organization of ribosome domains. First, the conventional head domain (helices 28 to 43) contains two distinct network communities (Fig 4C). Second, the long helix h44, which is considered to be a separate 3' minor domain, is structurally connected via RINGs to nodes in the head domain (Fig 4C, blue community). The remainder of the conventional head domain is occupied exclusively by nodes that belong to the red community, and this region includes some of the highest strength nodes observed in the network analysis.

The RING-based network analysis thus supports a physical model in which helices 28–40 at the 3' end of the 16S rRNA form what we call the outer-head domain, and helices 41–45 form the inner-head and spine domain (Fig 4C and 4D; red and blue communities, respectively). Based on a previous (and elegant) analysis of hinge motions in the small subunit, a region similar to our outer-head domain was previously identified as a distinct structural element in the head domain [10]. Nucleotides of the newly identified inner-head and spine domain have network connectivity suggestive of a cohesive structural entity that is distinct from the outer-head domain. The cohesive inner-head and spine domain spans nearly the entire length of the small ribosomal subunit (approximately 200 Å). The inner-head and spine domain is located at the intersubunit interface and makes extensive contacts within the active translational complex with the 50S subunit, tRNAs, mRNA, and translation factors. These external contacts involve coordinated rearrangements along the length of the inner-head and spine and, during translation, likely contribute to the experimentally observed cohesive nature of this long, structurally integrated domain.

Additional notable features are revealed by superimposing the network-based domain architecture on the three-dimensional structure [12] of the 16S rRNA (Fig 4D). In the RING-based model, the core constituents of each domain are compact, and notably more compact than the full RNA structure. Nodes are most dense in the center of the ribosome and in the head domain. Roughly one-third of the 16S rRNA does not contain RING-based nodes, and these node-less regions fall in the outer edges of the 30S subunit. RING analysis thus correctly identifies the functional core of the 30S ribosome subunit. Finally, the inner-head and spine domain (Fig 4C, blue) extends approximately 200 Å between the most distant nodes, revealing structural integration of inner regions of the conventional head domain and the h44 spine (Fig 4D). Correlated chemical probing thus supports a 4-domain model of the 30S subunit.

### Spc remodels intra-network communities and long-range interactions

In the absence of Spc, nucleotide A1111 is the largest node in the network; A1111 is engaged in extensive structural communication with other nodes within the red community (Fig 3B). Most structural communication in this no-Spc state is confined within the individual blue, red, yellow, and green communities. A1111 has the highest cross-community interactions of all nodes, and these are primarily with the blue community. A1111 and three other strong nodes of the red community (C1109, A1067, A1188) are part of a 3-helix junction region that is adjacent to the site of Spc binding (Fig 3D). Binding by Spc induced a large increase in the number of connections and enhanced the connections of A1111 both within the red community and across community boundaries to the blue community relative to the absence of Spc (Fig 3C, S3 Fig).

Binding by Spc also strengthened correlations throughout the inner-head and spine regions. In the presence of Spc, nodes in the inner-head community network became denser, and a modest remodeling of the long distance structural communication with the spine component of this community was observed (Fig 3D and 3E). The body (green) and platform (yellow) domains had relatively few correlations with other domains in the absence of Spc (Fig 3B). Upon binding by Spc, new incidences of strong structural communication were observed that radiate from the body domain. These correlations are centered at A563, which is the predominant node for structural communication between the green and yellow communities (Fig 3C and 3D).

### Cross-community interactions connect head swiveling and intersubunit rotation

**Fig 5:**
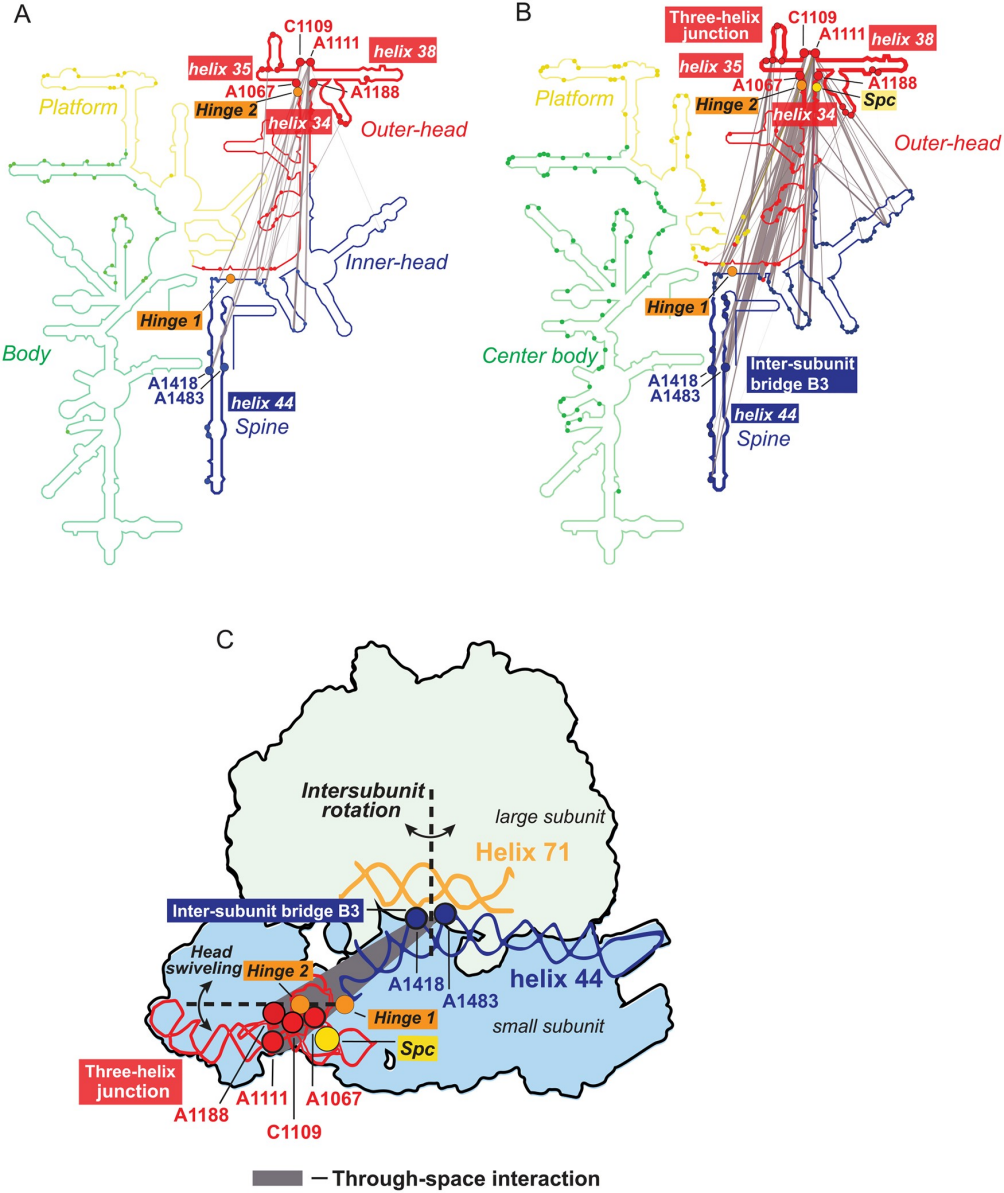
Cross-community interactions strengthened upon Spc binding. (A, B) Cross-community correlations connecting the outer-head (red) and inner-head and spine (blue) communities are shown as grey lines on secondary structure diagrams for experiments performed (A) without and (B) with Spc treatment. The strongest cross-community interactions link nodes in the outer-head domain at the h34-h35-h38 3-helix junction to nodes in helix 44 of the inner-head and spine domain. Orange spheres indicate the two hinges that allow head domain swiveling [10]. (C) Cross-community interactions visualized with respect to the three-dimensional structure of bacterial ribosome. The two dashed lines mark the axes for head swiveling (passing through hinges 1 and 2) and for intersubunit rotation (at bridge B3). Bridge B3 links helix 44 of 30S rRNA with helix 71 of the 23S rRNA of the large subunit. rRNA, ribosomal RNA; Spc, spectinomycin.

Next, we created maps of the 16S rRNA secondary structure showing all interaction edges between red and blue network communities in the absence and presence of Spc (Fig 5A). C1192, at the Spc binding site, is adjacent to the 3-helix junction formed by helices h34, h35, and h38 [12]. In the presence of Spc, there are long-distance connections between this 3-helix junction, located in the outer-head domain, and nucleotides A1418 and A1483 in h44 in the inner-head and spine domain. In the absence of Spc, these connections are relatively few in number and occur almost exclusively with A1418 in h44 (Fig 5A, gray lines). Upon binding by Spc, the strength and number of interactions involving the h44 nucleotides, especially A1483, increase. Numerous other interactions linking the outer-head domain with the inner-head and spine domain increase in number and become stronger upon binding by Spc (Figs 3E and 5B).

Strikingly, the cross-community interactions stabilized by Spc connect the two “hinges” that mediate large-scale motions in the 30S subunit. The 3-helix junction region is populated with strong nodes from the outer-head (red) community that overlap with one of the two hinge points (hinge 2, at C1066) [10] that mediate head swiveling (Fig 5A). The second hinge site for head rotation (hinge 1) is located in helix 28 at C1390, and the coordinated movement about these two hinges results in swiveling of the head domain (Fig 5C) [10]. At the other end of the cross-community interaction, A1418 and A1483 form the intersubunit bridge, termed B3, with helix 71 of the large subunit 23S rRNA. The axis of intersubunit rotation passes through bridge B3 (Fig 5C), and this structural feature is conserved in both prokaryotic and eukaryotic ribosomes [5,32,33]. The nucleotides that form this intersubunit bridge remain in contact during ratcheting of the two subunits during translation [5]. The major through-space cross-community interaction stabilized by Spc binding (Fig 5B) specifically connects the axis of head swiveling with the axis of intersubunit rotation (Fig 5C). This major consequence of Spc binding is invisible to standard chemical probing (Fig 2) and has not been detected in high-resolution structural studies. This cross-community connection, readily detected by single-molecule in-cell correlated chemical probing, reveals direct coordination between the two crucial pivot movements fundamental to ribosome-mediated translation.

## Discussion

We analyzed hundreds of occurrences of pair-wise through-space structural communication within the 30S subunit RNA structure in an unbiased way using RING-MaP single-molecule correlated chemical probing [17] in *E*. *coli* cells. We used network partitioning analysis to visualize nucleotides, domains, and communities that show interelement correlations (Fig 1). This network approach recapitulated the overall domain architecture of the 16S rRNA, correctly assigned elements of the central domain as defined by the secondary structure to the body domain (Fig 4B) and directly detected the structural and functional core of the 30S subunit (Fig 4D). Network analysis revealed that the conventionally assigned head domain contains two separate structural regions: an outer-head domain, which has been previously described as the structural core of the head domain [10], and a second community that connects the head domain with helix h44 to form a distinct structural unit that we call the inner-head and spine domain (Fig 4D).

Treating *E*. *coli* cells with the antibiotic Spc, a translocation inhibitor that disrupts proper movement of the small subunit head domain [8,10,12], induced a highly localized change in DMS reactivity of the small subunit RNA in ribosomes probed in cells (Fig 2A). In contrast, analyses of RING connectivities revealed that binding by Spc induced a dramatic strengthening of through-space interactions across the entire 16S rRNA (Figs 3 and 5). The antibiotic induced widespread enrichment of interactions in the outer-head, inner-head, and spine domains indicating that Spc binding stabilizes long-range interactions throughout the entire small subunit (Fig 3). Thus, the effect of Spc binding extends far beyond a localized effect at its direct binding site.

We identified strong through-space RNA interactions linking head swiveling with intersubunit rotation over distances spanning 95 Å (Fig 5). The principle pivot for head swiveling (hinge 2) lies in the h34-h35-h38 3-helix junction located in the outer-head (red) domain [1,2,10] (Fig 5A). Spc binds adjacent to this pivot point (Fig 5B and 5C). Although most Spc-resistance mutations occur proximal to this binding site, two (A1351C [34] and G1386A [35]) are distant from the binding site. Additionally Spc inhibits ultraviolet light–induced crosslinking between C934 and U1345 [36]. A1351, G1386, and U1345 all lie within the inner-head (blue) domain defined by our network analysis, corroborating our model that Spc (which binds in the outer-head domain, in red) has long-range effects on the 16S rRNA that span the outer-head and inner-head and spine domains. In the presence of Spc, the 3-helix junction in the outer-head domain had strong through-space RING interactions with the inner-head region, as well as with 2 nucleotides in the h44 spine region, A1418 and A1483 (Fig 5, gray lines). A1418 and A1483 form the intersubunit bridge B3 by interacting with helix 71 of the 23S rRNA, the anchoring pivot for intersubunit rotation [5,7,32,33,37]. Thus, our work reveals that head swiveling is directly correlated with intersubunit rotation, two motions essential for tRNA translocation, even though the sites of these two large-scale conformational changes are far apart in three-dimensional space.

**Fig 6:**
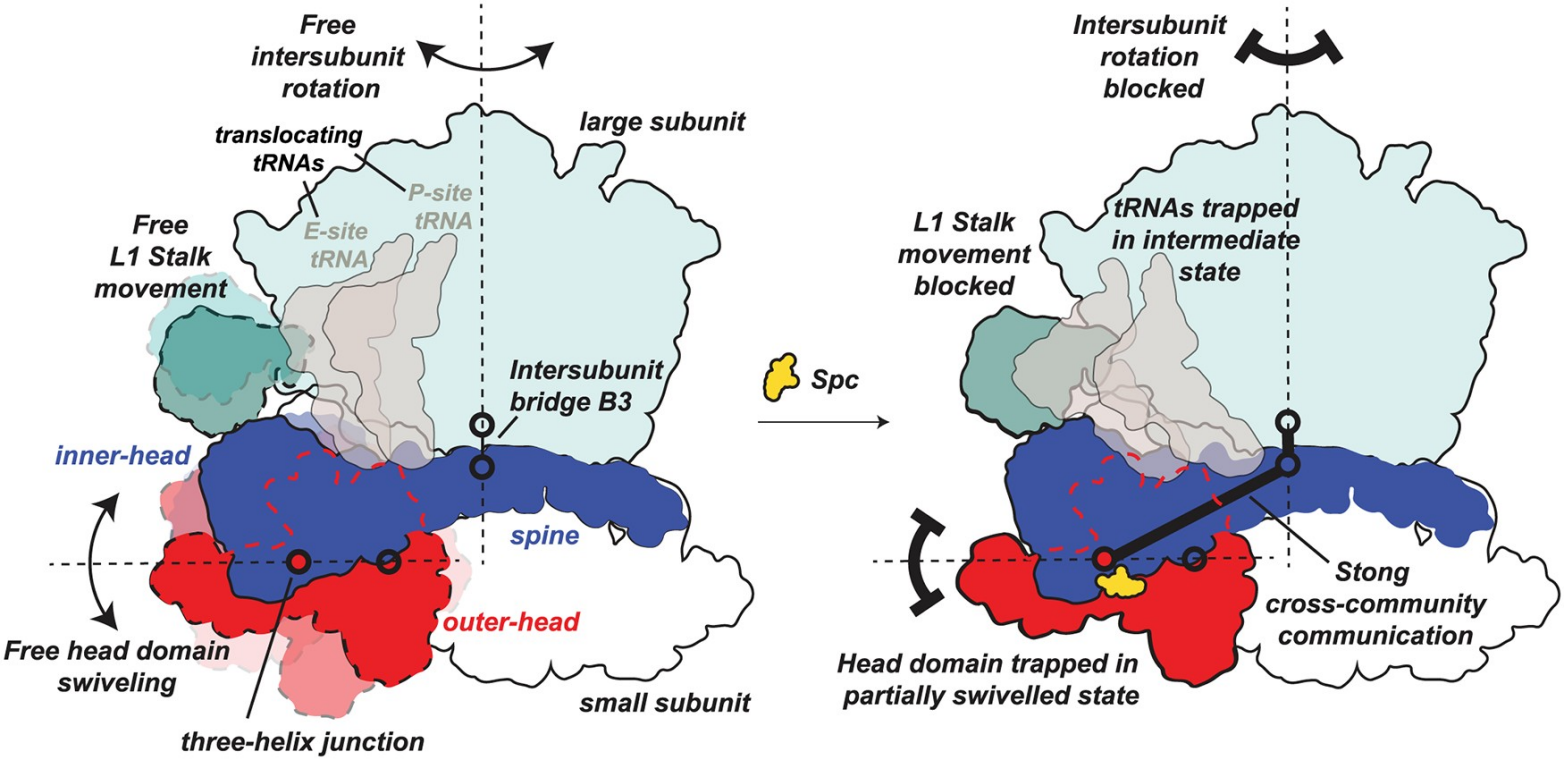
Effect of Spc on ribosome dynamics. Spc binding blocks free swiveling of the head domain and halts intersubunit rotation due to strengthened through-space interactions between pivot regions, thereby trapping the ribosome in a partially swiveled state. Black dashed lines indicate movements involving head swiveling and L1 stalk movement during translocation. Red dashed lines outline the portion of the outer-head domain concealed behind the inner-head domain. Spc, spectinomycin.

Together, these data support the following model for how Spc functions to inhibit translation. In the absence of Spc, through-space interactions between the pivot regions for head swiveling and intersubunit rotation are sufficiently weak to allow relatively fluid motions, including free head domain swiveling, intersubunit rotation, L1 stalk movement, and tRNA translocation [15] (Fig 6). In this state, several intersubunit bridges are dynamically disrupted and reformed allowing free intersubunit rotation. Head domain swiveling in particular involves disruption of intersubunit bridge B1a/b, an important RNA–protein and protein–protein bridge (connecting the universal small subunit protein uS13 to the central protuberance of the large subunit) [7,38]. Notably, the anchoring intersubunit bridge B3 remains intact throughout these movements [7]. Spc binding specifically strengthens interactions adjacent to the two hinge elements that allow head swiveling and creates strong interactions between hinge 2 and the intersubunit bridge B3 (Fig 5). Thus, Spc binding modulates intersubunit rotation at bridge B3 by strengthening through-space RNA–RNA interactions, which restricts the relative movement of the subunits during translocation (Fig 6). L1 stalk movement and tRNA translocation are also inhibited [39] (Fig 6), emphasizing that the observed through-space internucleotide communication we observe is likely mediated by contacts with other components of the ribosomal translation complex.

In the Spc-arrested state, head swiveling is no longer able to guide translocating tRNAs from the hybrid to the post-translocation positions [8,10,12]. Specifically, Spc traps the ribosomal complex when the tRNAs assume an intermediate conformation, with the 2 tRNA anticodon loops on the 30S A and P sites, and their respective acceptor arms in the 50S P and E sites [2,12] (Fig 6). Elongation factor-G normally enters the 30S A site and facilitates displacement of the tRNA anticodon to the P site [6,13,16] but cannot do so without the 30S P-site tRNA moving to the E site, a movement guided by head swiveling [8]. Critically, these external contacts of the small subunit RNA—involving the large subunit, tRNAs, domain IV of elongation factor-G, and other intersubunit bridges [7]—are mediated by the inner-head and spine domain, which our study reveals is a structurally integrated entity (Fig 6).

Structural and biophysical studies also indicate that Spc traps the head domain in a partially swiveled state [12] that contains tRNAs in a compacted conformation stabilized in an intermediate state of translocation [5,15]. This compaction occurs because the elbow regions of tRNAs are blocked by the 50S subunit L1 stalk, which normally moves to allow tRNA translocation on the 50S at a coordinated rate with respect to head swiveling (Fig 6) [39]. This restricted tRNA movement in the Spc-bound state prevents L1 stalk movement, resulting in no intersubunit rotation (Fig 6) [1,2]. Our model specifically depicts the distance and strength of this interaction (Fig 6). Thus, Spc binding locks the gears (intersubunit rotation and swiveling) of ribosome dynamics by effectively tying two distinct pivots to each other.

RING-MaP, a single-molecule experiment, revealed extensive long-range structural communication throughout the small subunit of the ribosome in living cells. RING analysis enabled discovery of previously unobserved rRNA domain structure and through-space interactions. Analysis of Spc binding revealed how a small molecule can bind to and block the function of a large megadalton complex and suggests why it has proven so difficult to develop new ribosome-targeting antibiotics that function by novel mechanisms. This work reveals that Spc does not function by simple occlusion, local stabilization, or trapping but instead alters structural communication networks throughout the ribosome. Targeting RNA–RNA interactions, such as those that create the cross-domain connection we observe between h34-h35-h38 3-helix junction and the B3 bridge (Figs 4 and 5) might be a compelling drug-development strategy. We anticipate that single-molecule correlated probing will reveal new principles of large RNA domain and RNA-protein organization and structural communication when applied to functional motifs in viral RNAs, mRNAs, and long noncoding RNAs.

## Methods

### Bacterial cell growth and antibiotic treatment

Overnight cultures (2 mL) were added to 48 mL of LB media. Cells were incubated with shaking until the culture reached an OD_600_ of approximately 0.5 (~30 min), at which time cells were collected by centrifugation. For antibiotic treated cells, 5.55 mL of a 187.5 μg/mL Rif solution were added, and cells were incubated with shaking for 10 min. Following incubation, 27 mL of each culture were transferred to a new culture flask. To each culture, either 3 mL of water or 3 mL of a 494 μg/mL of Spc were added. Cultures were incubated with shaking for 10 min. Cells were pelleted in 25-mL aliquots by centrifugation at 4,000*g* for 20 min. Supernatants were discarded, and the cell pellet was resuspended in 200 μL of folding buffer containing 300 mM cacodylate (pH 7.0), 200 mM potassium acetate (pH 7.0), and 10 mM MgCl_2_ and incubated at 37 °C for 5 min. Note that all +Spc samples were also pretreated with Rif.

### DMS treatment and purification of rRNA

Aliquots (90 μL) of cells were added to 10 μL DMS (1:5 dilution in neat ethanol) ([+] reaction) or 10 μL neat ethanol ([–] reaction) and incubated at 37 °C for 6 min. Following incubation, an equal volume (100 μL) of neat 2-mercaptoethanol was added to quench the DMS. To each sample, 1 mL of TRIzol (Invitrogen, Carlsbad, CA) was added, and the reaction tubes were incubated at room temperature. After 5 min, 200 μL of cold chloroform was added, and tubes were shaken vigorously by hand for 15 s. Samples were incubated at room temperature for 2 to 3 min. Tubes were centrifuged at 12,000*g* for 15 min at 4 °C. The aqueous (upper) layer was transferred to a new tube, and 1.1 mL isopropanol was added. Reactions were incubated at −20 °C for 30 min and then centrifuged at 15,000*g* for 30 min at 4 °C. The supernatant was discarded, and pellets were carefully washed twice with 500 μL 80% ethanol, centrifuging 5 min at 15,000*g* between washes. Following the washes, the supernatant was discarded, and pellets were dried in air for 5 min. After resuspension in RNase-free water, samples were then treated with DNase I (Ambion, Carlsbad, CA) to remove any contaminating genomic DNA and subjected to affinity purification (RNeasy Mini Kit, Qiagen, Germantown, MD).

### Reverse transcription under MaP conditions

To 700 ng of RNA was added 200 ng of random 9-mer primer, 2 μL of 10 mM dNTPs (Fermentas, Waltham, MA), and water to a final volume of 10 μL. Primers were annealed at 65 °C for 5 min followed by incubation at 4 °C for 2 min. Next, 9 μL of buffer master mix (2 μL of 500 mM Tris [pH 8.0], 750 mM KCl, and 100 mM DTT; 2.76 μL water and 4 μL 5 M betaine (Sigma, St Louis, MO); and 0.24 μL 500 mM MnCl_2_) was added to the annealed reaction mix. These conditions incorporate high concentrations of betaine and a primer annealing protocol that yield improved and efficient reverse transcription of DMS-modified products [40]. After incubation at 25 °C for 2 min, 1 μL of SuperScript II (Invitrogen, Carlsbad, CA) was added, and samples were incubated according to a stepped primer extension protocol: 25 °C for 10 min followed by 42 °C for 90 min, and then 10 cycles of 2 min at 50 °C and 2 min at 42 °C. The reverse transcriptase enzyme was inactivated by incubating the samples at 70 °C for 10 min. Following primer extension, cDNA products were purified (RNAclean beads, 1.8 bead to sample ratio; Beckman Coulter, Indianapolis, IN). Purified RNA was eluted from the beads in 68 μL nuclease-free water and converted to double-stranded DNA (dsDNA) using a second-strand synthesis enzyme mix (NEB, Ipswitch, MA). Following second-strand synthesis, dsDNA was purified (AmpureXP beads, 0.7:1 bead to sample ratio; Beckman Coulter, Indianapolis, IN). Product sizes following second-strand synthesis were analyzed (Agilent Bioanalyzer 2100).

### Library preparation and sequencing

For library preparation, 1 ng of each second-strand synthesis product was used to create libraries for sequencing (NexteraXT, Illumina, San Diego, CA); final libraries were size selected (AmpureXP beads, using a 0.5:1 bead to sample ratio; Beckman Coulter, Indianapolis, IN) and quantified (Agilent Bioanalyzer 2100 and QuBit high-sensitivity dsDNA assays). Sequencing was performed on an Illumina NextSeq 500 system with a loading concentration of 1.4 pM, yielding approximately 400 million reads.

### Data processing and alignment

Adapter sequences were removed from raw FASTQ files using the program *scythe* (version 0.991; available at https://github.com/vsbuffalo/scythe) with default parameters [41]. Reads were then trimmed for quality using *sickle* (version 1.33; available at https://github.com/najoshi/sickle) in paired-end mode with a Phred quality cutoff of 20 and a minimum length of 20 [42]. Only pairs for which both mates passed filtering were used in downstream stages. Following adapter removal and quality trimming, *ShapeMapper* (version 1.2) was used to map the processed FASTQ files to the 16S and 23S sequences [26,43]. No further quality trimming was performed during the quality trimming stage in *ShapeMapper*. During the read alignment stage, 2 additional flags in Bowtie2 were used to force concordant alignments: “—no-discordant” and “—no-mixed.” The following options were changed from the defaults to optimize for long insert sizes: “maxInsertSize = 1200,” “minMapQual = 30,” and “minPhredToCount = 30.”

### Analysis framework for randomly primed RING-MaP reads

The previously reported RING analysis approach [17] required that all sequencing reads be stored in computer memory in order to perform association analyses. This approach is appropriate for small RNAs but impractical for RNAs as long as the 16S rRNA. Rather than retaining all sequencing reads in memory, it is possible to create a simplified representation of alignment and mutation location information (S1 Fig). This representation is a two-dimensional array with each element containing a contingency table of the counts and kinds of interactions (S1 Fig). Information about pairwise observations of mutations within each read can thus be counted and stored independently. Using this strategy, the total amount of memory needed for analysis depends only on the RNA length and not on the number of sequencing reads. During analysis of sequencing data, only reads that meet Phred quality cutoffs were included. Since interactions are stored as *i-j* interaction pairs, long stretches of incomplete information (such as gaps between sequencing reads relative to the reference sequence) were allowed, with each *i-j* point in the matrix representing the contingency table for all reads that contained both nucleotides *i* and *j*. Using the contingency table, a Yates chi-squared statistic and Pearson correlation (phi) was calculated (S1 Fig). Correlations with Yates chi-squared values above 20 were considered significant. Based on this threshold for chi-squared statistics, the probability that correlated nucleotides were independent was less than 10^−5^.

In paired-end sequencing, both ends of the DNA library are sequenced even though they may be separated by several hundred nucleotides. Modern sequencing platforms keep paired reads “together,” effectively allowing detection of interactions at a distance up to the size of the inserts of the sequencing library. In this work, large DNA fragments were selected, and sequencing libraries were constructed with insert sizes between 500 and 700 nucleotides. Approximately 50,000 reads between 2 locations of the RNA were needed to reliably detect correlated interactions. At this sequencing depth, for each of our samples, we can reliably detect interactions between nucleotides that are separated by 450 to 650 nucleotides in sequence space with the specific number depending on biases resulting from random priming (S1 Fig).

### Correlation analysis of randomly primed reads

The “mutation strings” files from the *ShapeMapper* pipeline were used as input for randomly primed correlation analysis; these files contain a simplified representation of the read alignment location, mutation locations, and sequencing instrument quality calls. A square matrix was constructed with a size equal to the length of the aligned RNA (S1 Fig). Each element (P) contains a 2 × 2 contingency matrix representing the possible outcomes in comparing 2 nucleotides (S1 Fig). In each read, all *i*-*j* combinations of nucleotides were used to index the storage matrix. Mutations (scored as 1) and matching nucleotides (scored as 0) were used to index the contingency table. Only nucleotides with a phred score above 30 were counted. We also excluded 26 residues that showed a high background mutation rate (in the non–DMS-treated sample), most of which correspond to known single nucleotide polymorphisms in the *E*. *coli* 16S rRNA gene. After all reads were processed, the storage matrix contained an easily indexed representation of the entire sequencing dataset, and each *i-j* element in the matrix contained a snapshot of all the reads that span nucleotides *i* and *j*. The total number of times nucleotides *i* and *j* were read together (N_*i*,*j*_) is the sum of all the elements in the contingency table. Next, each *i* < *j* pair in the read storage matrix was tested for significance using the Yates chi-squared test with a significance criterion of 20 (S1 Fig); the strength of the correlation was measured using the Pearson *r* metric. Correlations from the two 2 biological replicates, performed for each condition, were pooled by requiring that a correlation pair occur in both replicates to be included (S2 Fig).

### Network analysis of correlations in the 16S rRNA

Correlation values from the +Rif and +Spc samples were analyzed using the network visualization software *Gephi* (version 0.9.2; available at https://gephi.org) (S1 Fig) [30]. A network diagram was drawn as an “undirected graph,” which produces a network of nodes with connecting edges such that all edges are bidirectional. Filtering was used to restrict the network population requiring that (i) the strength of the correlation must be greater than 0.015 and that (ii) each node must have at least 3 connections (k-core = 3). Nucleotides were treated as network nodes, and edges depict correlation strength between connected 2 nodes. Network diagrams were arranged such that nodes linked by stronger connecting weights attract each other, while nodes with weaker connecting weights are pushed apart (Force Atlas option). Node color and size and edge color and thickness were set (Ranking Module, using Degree as the ranking parameter). In an undirected graph, the degree of a node is simply the sum of all its edges. Communities were detected by Modularity, using the Louvian method [44]; graph modularity was calculated with sensitivity setting of 1.0. This community detection algorithm creates a Modularity Class for each node, which was used to partition the network into communities, represented by different colors.

To visualize the network graph on the structure of the ribosome, the Average weighted degree parameter was used to generate a list of nodes ranked by the sum of the total weight (correlation strength) of all its edges. Nodes were categorized as strong (greater than 0.25), medium (between 0.1 and 0.25), or weak (lower than 0.1) based on average weighted degree values. Nucleotides corresponding to these nodes are represented as spheres (large, medium, or small) on the structure of the ribosomal small subunit (PDB 4v56) [6]. Network edges were ranked by correlation strength, and categorized as strong (75th percentile), medium (50th–75th percentile), or weak (below 50th percentile). Strong edges are represented on the three-dimensional structure of the ribosomal small subunit as colored lines. Small ribosomal subunit domains by network community data were defined by including regions occupied by high strength nodes in both the absence and presence of Spc. Nodes from the more extensive +Spc network graph were then superimposed on the network defined domains (Fig 3).

## Supporting information

S1 Fig Algorithmic innovations to enable RING-MaP analysis of large-scale interactions using randomly primed reverse transcription. (A) Data storage matrix used to show counts of within-read interactions. (B) Contingency table for each data storage element (P). (C) Equations for calculating significance of each interaction performed after read counting using Yates chi-squared test. Correlation strength was calculated using the Pearson *r* metric. (D) Detection interval for DMS-modified 16S rRNA using the optimized cDNA synthesis protocol. The effective maximum detection interval for correlation analysis requires approximately 50,000 reads. This interval is enclosed with a dashed line.
(TIF)

S2 Fig RING-MaP correlations measured for replicate in-cell states of the small ribosomal subunit. (A) Correlations from 2 biological replicates were merged; only those that occurred in both replicates were retained. (B) RING-MaP correlations for merged replicates as a function of cellular state. Conventional domain boundaries for the 16S rRNA are indicated. Base pairs (top) present in the structure established by covariation analysis are shown as gray arcs. Correlations for each of the merged datasets for the 3 in-cell conditions are shown as red and blue arcs for positive and negative correlations, respectively. The underlying data for this figure are available at: https://doi.org/10.6084/m9.figshare.9252995.v1.
(TIF)

S3 Fig In-cell binding by Spc increases numbers of and strengths of correlations. (A) Edges present in +Spc network diagram colored by correlation with the same edges in the +Rif network. (B) Correlations strengthened in the presence of Spc relative to the +Rif network. Correlation strength is illustrated by edge thickness. The underlying data for this figure are available at: https://doi.org/10.6084/m9.figshare.9252995.v1.
(TIF)

## Abbreviations

DMS: dimethyl sulfate
dsDNA: double-stranded DNA
MaP: mutational profiling
PDB: Protein Data Bank
Rif: rifampicin
RING: RNA interaction group
RING-MaP: RNA interaction groups analyzed by mutational profiling
rRNA: ribosomal RNA
Spc: spectinomycin
uS13: universal small subunit protein S13
