# Supplementary figures and images for: Single-molecule correlated chemical probing reveals large-scale structural communication in the ribosome and the mechanism of the antibiotic spectinomycin in living cells
Source: PLoS Biol. 2019 Sep 5;17(9):e3000393. doi: 10.1371/journal.pbio.3000393 (PMC6748448; doi:10.1371/journal.pbio.3000393)

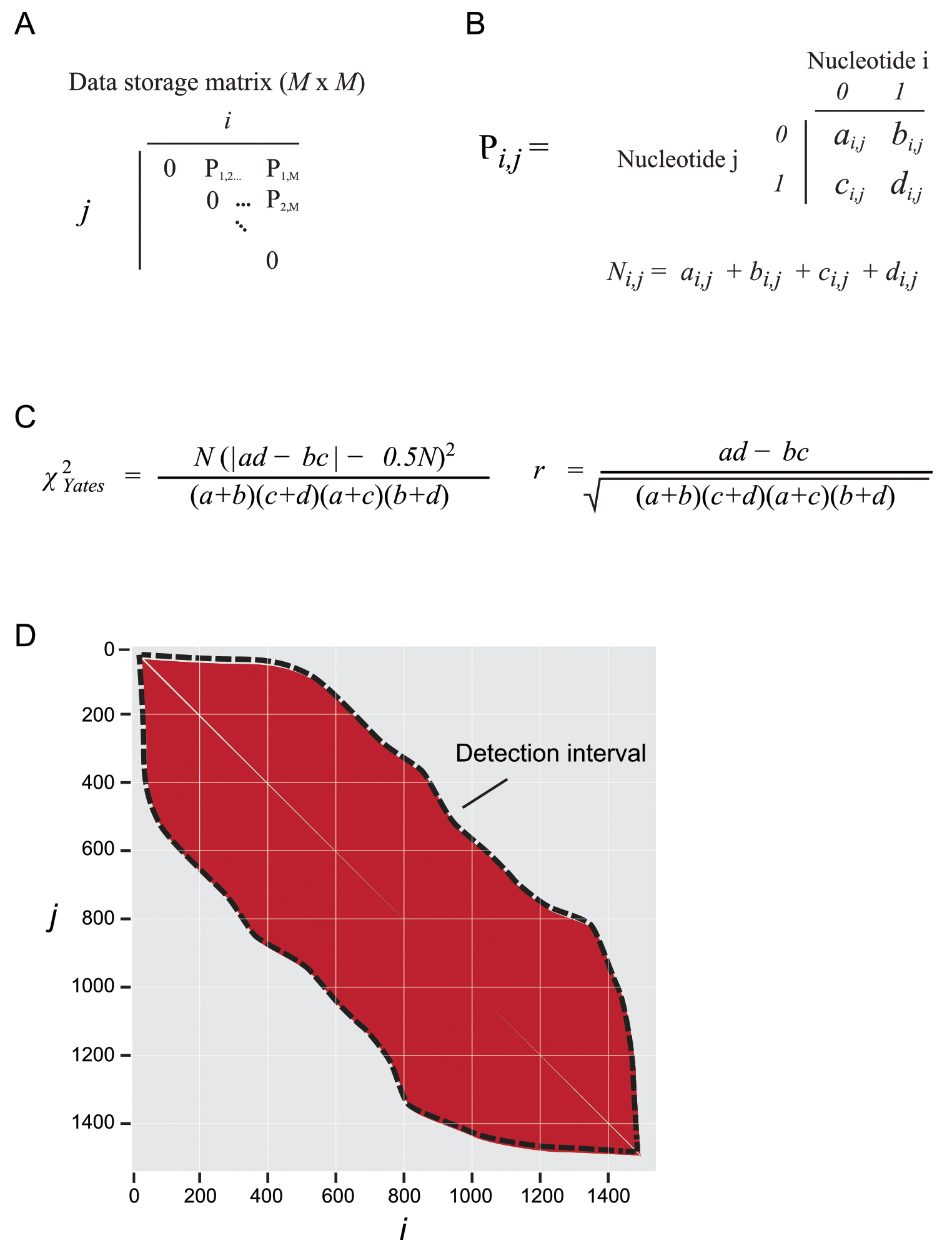

Supplement: S1 Fig — (A) Data storage matrix used to show counts of within-read interactions. (B) Contingency table for each data storage element (P). (C) Equations for calculating significance of each interaction performed after read counting using Yates chi-squared test. Correlation strength was calculated using the Pearson r metric. (D) Detection interval for DMS-modified 16S rRNA using the optimized cDNA synthesis protocol. The effective maximum detection interval for correlation analysis requires approximately 50,000 reads. This interval is enclosed with a dashed line. (TIF) [file pbio.3000393.s001.tif]

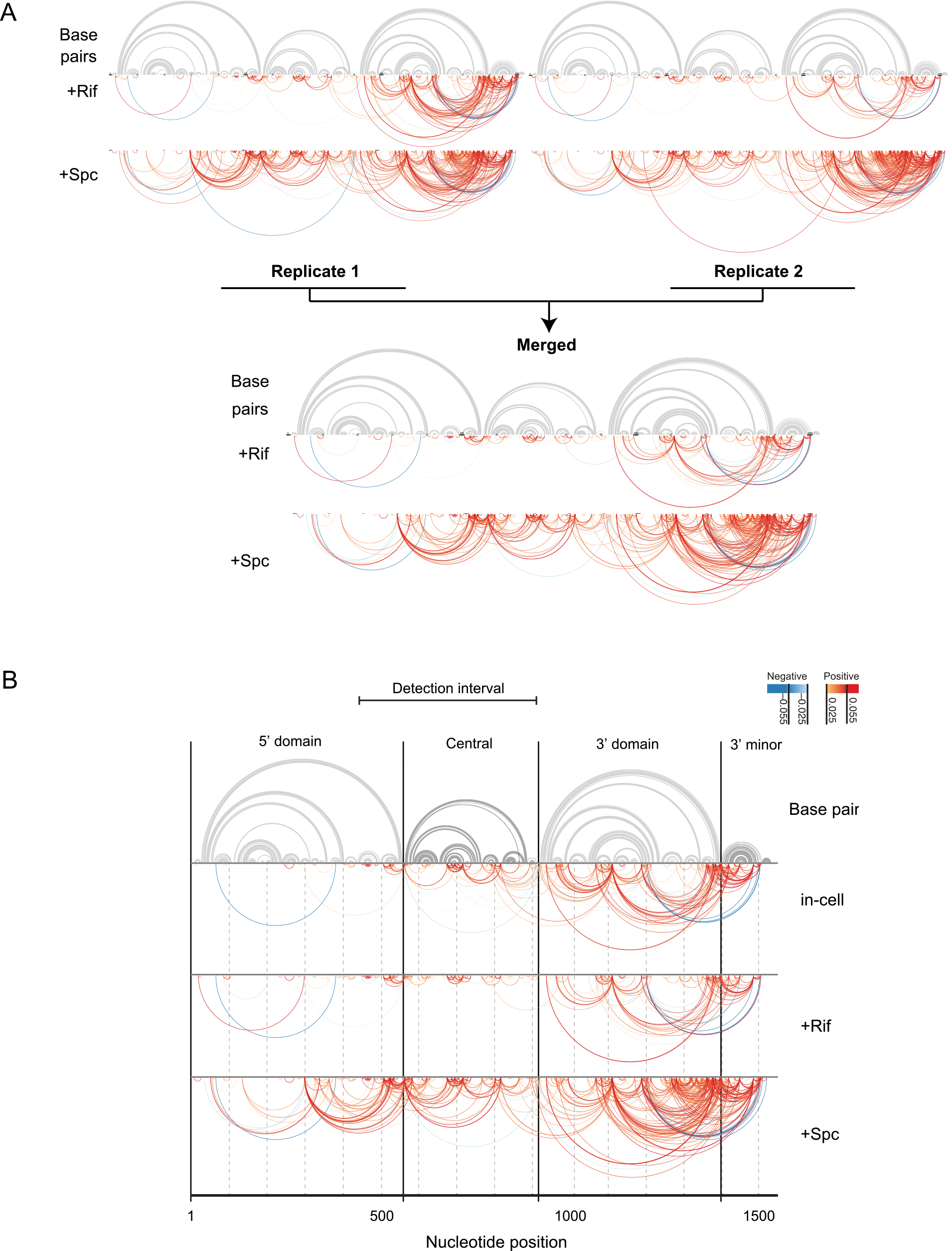

Supplement: S2 Fig — (A) Correlations from 2 biological replicates were merged; only those that occurred in both replicates were retained. (B) RING-MaP correlations for merged replicates as a function of cellular state. Conventional domain boundaries for the 16S rRNA are indicated. Base pairs (top) present in the structure established by covariation analysis are shown as gray arcs. Correlations for each of the merged datasets for the 3 in-cell conditions are shown as red and blue arcs for positive and negative correlations, respectively. The underlying data for this figure are available at: https://doi.org/10.6084/m9.figshare.9252995.v1. (TIF) [file pbio.3000393.s002.tif]

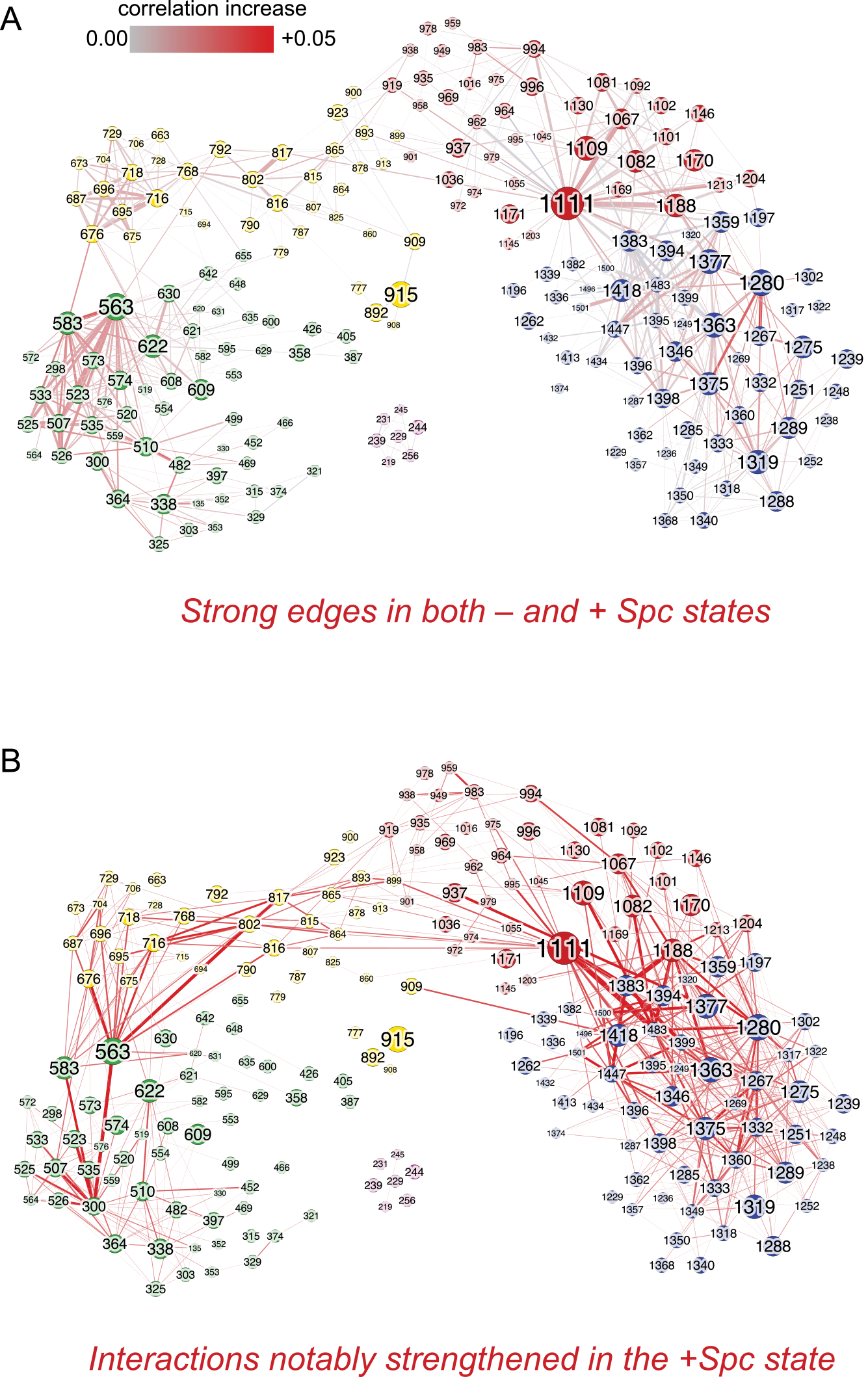

Supplement: S3 Fig — (A) Edges present in +Spc network diagram colored by correlation with the same edges in the +Rif network. (B) Correlations strengthened in the presence of Spc relative to the +Rif network. Correlation strength is illustrated by edge thickness. The underlying data for this figure are available at: https://doi.org/10.6084/m9.figshare.9252995.v1. (TIF) [file pbio.3000393.s003.tif]
